# Feasibility of Modeling Orthogonal Frequency-Division Multiplexing Communication Signals with Unsupervised Generative Adversarial Network

**DOI:** 10.6028/jres.126.046

**Published:** 2022-02-14

**Authors:** Jack Sklar, Adam Wunderlich

**Affiliations:** 1 Communications Technology Laboratory, National Institute of Standards and Technology, Boulder, CO 80305, USA

**Keywords:** generative adversarial network (GAN), machine-learning, RF data sets, time series

## Abstract

High-quality recordings of radio frequency (RF) emissions from commercial communication hardware in realistic environments are often needed to develop and assess spectrum-sharing technologies and practices, e.g., for training and testing spectrum sensing algorithms and for interference testing. Unfortunately, the time-consuming, expensive nature of such data collections together with data-sharing restrictions pose significant challenges that limit data set availability. Furthermore, developing accurate models of real-world RF emissions from first principles is often very difficult because system parameters and implementation details are at best only partially known, and complex system dynamics are difficult to characterize. Hence, there is a need for flexible, data-driven methods that can leverage existing data sets to synthesize additional similar waveforms. One promising machine-learning approach is unsupervised deep generative modeling with generative adversarial networks (GANs). To date, GANs for RF communication signals have not been studied thoroughly. In this paper, we present the first in-depth investigation of generated signal fidelity for GANs trained with baseband orthogonal frequency-division multiplexing (OFDM) signals, where each subcarrier is digitally modulated with quadrature amplitude modulation (QAM). Building on prior GAN methods, we developed two novel GAN models and evaluated their performance using simulated data sets with known ground truth. Specifically, we investigated model performance with respect to increasing data set complexity over a range of OFDM parameters and conditions, including fading channels. The findings presented here inform the feasibility of use cases and provide a foundation for further investigations into deep generative models for RF communication signals.

## 1 Introduction

To aid the development of spectrum-sharing technologies, practices, and policies [1, 2] for radio frequency (RF) communication systems, it is necessary to characterize emissions in the band of interest, assess spectrum-sensing performance, and evaluate interference between heterogeneous systems [2-6]. One issue complicating such studies is the time and expense required to collect high-quality, real-world recordings of RF in-phase and quadrature (I/Q) signals; see [7-9] for examples.

Although early-stage development can be executed with simulated data, it is very challenging to develop accurate models of RF I/Q time series for deployed commercial off-the-shelf (COTS) communication systems from first principles. Specifically, in typical measurement scenarios, system parameters and implementation details are at best only partially known, and complex system dynamics are difficult to characterize. For example, with limited information, it is difficult to accurately model effects due to the analog RF front-end, power control and scheduling dynamics, traffic loading, and wireless propagation [3].

Considering the difficulties of realistic modeling of I/Q signals from "black-box" systems, there is a need for flexible, data-driven alternatives that can leverage existing data sets. Unfortunately, there is currently no established state-of-the-art modeling approach for this problem, and experimental testing often relies on idealistic, synthetic signals, *e.g.*, Refs. [10-12]. One way to characterize a data set with unknown properties is through the use of unsupervised deep generative modeling.

Deep generative models utilize a deep neural network to produce samples from a high-dimensional target distribution defined by a training set. In recent years, deep generative models, and most notably, generative adversarial networks (GANs), have drawn a great deal of attention in the machine-learning community and have progressed rapidly; *e.g.*, see Refs. [13-17] for reviews. Namely, GANs and other deep generative models have been used to successfully synthesize and process realistic images, speech, and video. Because deep generative models can be trained in an unsupervised manner, they are a potentially flexible and powerful approach for data-driven modeling of RF I/Q time series with unknown, complex attributes.

To date, most work on deep generative models has focused on computer vision applications with images [13-17], while time series have received less attention. Nonetheless, there have been several papers proposing generative models for time series, primarily for audio applications. Examples of non-GAN deep generative models for audio time series include WaveNet [18], an autoregressive model, and MusicVAE [19], which uses a variational autoencoder (VAE).

Several time-series GAN models leverage prior work on GANs for images by training the generator to produce an image-domain time-frequency representation, such as a spectrogram, which is then mapped into a time series. Examples of models employing this approach include SpecGAN [20], MelGAN [21], TSGAN [22], GANSynth [23], and TiFGAN [24]. Additional approaches were compared by Nistal *et al.* [25].

Alternatively, there have been some papers proposing GANs that directly model time series. One class of methods includes architectures based on recurrent neural networks (RNNs), such as long short-term memory (LSTM), *e.g.*, Refs. [26, 27]. Methods based on convolutional neural networks (CNNs) include WaveGAN [20], which employs a flattened version of the popular DCGAN model [28], and QuantGAN [29], which uses temporal convolutional networks (TCNs) [30].

In the field of wireless communications, there have been some recent efforts to apply GANs. Examples include GANs for data augmentation for signal classification [31, 32], wireless channel modeling [33], anomaly detection [34], and adversarial attacks on communication systems [35, 36]. Due to their focus on use case performance, *e.g.*, signal classification accuracy, none of the aforementioned works presents comprehensive evaluations of generated signal fidelity, nor do they compare their models to other approaches. Furthermore, these prior works did not investigate how their models performed with respect to increasing data set complexity and signal length. As such, they provide little insight into generative model effectiveness and associated performance limitations.

In this paper, we present the first in-depth investigation of generated signal fidelity for GANs applied to unsupervised modeling of baseband I/Q orthogonal frequency-division multiplexing (OFDM) signals, where each subcarrier is digitally modulated with quadrature amplitude modulation (QAM) [37, 38]; see Sec. 2 for background. Because OFDM is a commonly used modulation and encoding scheme for digital transmission that is used in cellular networks and wireless local area networks (WLANs) [39, 40], investigations with OFDM signals provide a strong basis for generative modeling of communication signals. Using synthetic data with known ground truth, we investigated model performance over a range of OFDM parameters and conditions, including fading channels.

The findings presented here with synthetic data are anticipated to provide a foundation for future investigations into generative models trained with real-world recordings of RF time series and other types of communications signals. In particular, we are primarily interested in using GANs to generate waveforms for interference testing as well as for data obfuscation. By focusing on experiments with simulated data, we are able to gain important early-stage insights into model effectiveness that would not otherwise be possible. Namely, the ease of creating unlimited amounts of synthetic data and the ability to control OFDM parameters allow us to investigate the ability of models to handle increasing data set complexity. Moreover, because synthetic data enable straightforward symbol demodulation, it is possible to directly evaluate QAM symbol constellation fidelity. These findings indicate which use cases that are feasible for unsupervised generative models as well as those requiring additional advances.

## 2 Background: Digital Communication Signals

We review relevant aspects of digital modulation and wireless channel models in this section. Further background can be found in textbooks on wireless communications, *e.g.*, Refs. [37, 38].

### 2.1 Baseband Signal Representation

For a typical narrowband wireless communication system, where the signal bandwidth is much smaller than the carrier frequency, *f_c_*, the transmitted bandpass signal can be expressed in the alternative forms

*x*(*t*) = *r*(*t*) cos(2*πf_c_t* + ⲫ(*t*)) (1a)

 = *Re*{*s*(*t*) exp(*j*2*πf_c_t*)} (1b)

 = *Re*{[*I*(*t*) + *jQ*(*t*)] exp(*j*2*πf_c_t*)]} (1c)

 = *I*(*t*) cos(2*πf_c_t*) - *Q*(*t*) sin(2*πf_c_t*). (1d)

Above, *r*(*t*) ≥0 and *ⲫ*(*t*) are the time-varying amplitude and phase, respectively, *j*^2^ =-1, and *Re*{·} denotes the real part of a complex number. The quantity, *s*(*t*) = *r*(*t*) exp[*jⲫ*(*t*)], called the baseband or lowpass representation of the signal, can be decomposed as *s*(*t*) = *I*(*t*) + *jQ*(*t*), where the real and imaginary components are called the in-phase and quadrature (I/Q) components of the signal, respectively [37].

### 2.2 Quadrature Amplitude Modulation

Digital modulation maps a sequence of data bits into a physical waveform. A commonly used digital modulation method, called quadrature amplitude modulation (QAM), can be interpreted as a combination of digital-amplitude and digital-phase modulation [37]. Namely, for M-ary QAM (M-QAM), the transmitted bandpass signal for the *m*th symbol takes the form

*x_m_*(*t*) = *A_m_g_T_* (*t*) cos(2*π f_c_t*) + *B_m_g_T_* (*t*) sin(2*πf_c_t*) (2a)

= *Re*{*C_m_g_T_* (*t*) exp(*j*2*πf_c_t*)} (2b)

for *m* = 1, 2, *. . ., M*, where *A_m_* and *B_m_* are real-valued amplitude levels, *C_m_* = *A_m_* - *jB_m_* is the corresponding complex-valued amplitude, and *g_T_* (*t*) is a real-valued pulse-shape function of duration *T* [37]. The set of complex amplitudes, *C_m_*, can be visualized as points in an I/Q-space constellation diagram, which is typically a rectangular grid with *M* equal to a power of two. In this case, each symbol represents *k* = log_2_(*M*) bits. Gray encoding, in which adjacent symbols differ by only one bit, is a preferred method to map *k* bits to a symbol [37].

### 2.3 Orthogonal Frequency-Division Multiplexing

Modern wireless communication systems aim to achieve high data rates across wireless channels with time-varying, frequency-selective distortions. One digital modulation scheme that was designed for such conditions is orthogonal frequency-division multiplexing (OFDM) [37, 38]. OFDM converts a high-rate data sequence into multiple low-rate data sequences that are transmitted over parallel, narrowband channels that can be easily corrected (equalized) for channel distortions [38]. OFDM is used by many modern communication systems, including cellular networks [39] and wireless local area networks (WLANs) [40].

OFDM modulates symbols in parallel across *N_sc_* subcarriers. Specifically, a discrete time, baseband OFDM signal for a single symbol period, *T_s_*, has the (unnormalized) form

$s(t_{k})=\sum_{n=0}^{N_{SC}-1}C_{n}\exp(j2\pi nk/N),$ (3)

where *t_k_* = *kT_s_/N_sc_* (for *k* = 1, 2, *. . ., N_sc_*) are discrete time samples, and *C_n_* is the complex-valued symbol for the nth subcarrier [37, 38]. When the symbols are drawn from a QAM constellation, the resulting scheme is called OFDM/QAM, where classic OFDM/QAM uses a rectangular pulse shape [41]. Because Eq. (3) is simply an inverse discrete Fourier transform (DFT) on the block of *N_sc_* transmit symbols, OFDM is usually implemented with the inverse fast Fourier transform (FFT) algorithm [37, 38].

Multipath propagation in wireless channels causes delay dispersion and frequency-selective fading [38]. Delay dispersion gives rise to intersymbol interference (ISI). Also, in the case of multiple-carrier systems, such as OFDM, delay dispersion leads to a loss of orthogonality between subcarriers, resulting in intercarrier interference (ICI) [38, Sec. 19.7] [42]. Standard implementations of OFDM/QAM alleviate both ISI and ICI by using a special type of guard interval called a cyclic prefix [37, 38]. A cyclic prefix is a copy of the last part of the symbol of duration *T_cp_* that is prepended to the symbol, so that the total symbol duration is *T_cp_* + *T_s_*. At the receiver, the cyclic prefix, which may be corrupted by delay dispersion, is discarded. Assuming that the channel is static for the duration of an OFDM symbol and that the cyclic prefix is long enough, the effects of delay dispersion are alleviated, reducing ISI and ICI. The resulting OFDM system can be modeled by a set of parallel nondispersive, fading channels, each with its own complex-valued transfer coefficient. Consequently, equalization (correction) of the OFDM symbol for channel effects is very simple and can be carried out with element-wise division by the transfer function in the frequency (subcarrier) domain. The frequency-domain transfer function is typically estimated by using pilot symbols [38].

### 2.4 Multipath Channel Models

Multipath propagation in wireless channels results in frequency-selective fading; *i.e.*, the transfer function of the channel varies in frequency. A widely used class of statistical models for multipath propagation relies on the wide-sense stationary uncorrelated scatterers (WSSUS) assumption [38, 43]. A WSSUS channel can be represented as a tapped-delay-line filter, where the coefficients of each tap vary in time. Namely, the channel impulse response is

$h(t,\tau )=\sum_{i=1}^{N}C_{i}(t)\delta (\tau -\tau_{i}),$ (4)

where *N* is the number of multipath components, *c_i_*(*t*) are time-dependent coefficients, *δ* (*t*) is the Dirac delta function, and *τ_i_* is the time delay of the ith tap [38]. The most commonly used version of this model, the *N*-tap Rayleigh fading model, takes the coefficients, *c_i_*(*t*), to be zero-mean complex Gaussian random processes with autocorrelation functions determined by associated Doppler spectra, *e.g.*, the classical Doppler spectrum [38]. A well-known collection of *N*-tap Rayleigh fading models is specified in the Third-Generation Partnership Project (3GPP) cellular standard [44, Annex B.2]. We used these models to simulate the effects of multipath propagation in the fading channel experiment presented in Sec. 8.3.

## 3 Background: Generative Adversarial Networks

Here, we summarize key features of GAN models and Wasserstein loss with gradient penalty, the loss function that we used for training our models. Familiarity with the fundamentals of deep neural networks is assumed. For general background on deep learning, see the textbook by Goodfellow *et al.* [45].

### 3.1 Generative Adversarial Networks

A GAN consists of two neural networks, a generator, *G*, and a discriminator, *D*. The generator is trained to generate samples from a target data distribution, and the discriminator attempts to distinguish between generated and target data samples. Specifically, the generator, *G*(*z*), learns to map latent vectors, *z*, drawn from a multidimensional Gaussian distribution, *p_Z_*(*z*), to the generator distribution, *p_g_*. The discriminator maps sample data, *x*, to *D*(*x*), representing the probability that a sample belongs to the target distribution, *p_d_*.

In the original GAN formulation [46], the generator and discriminator compete against each other in the form of a zero-sum game, although it was soon discovered that the original GAN model can suffer from training instabilities, such as diverging loss and mode collapse. Subsequently, a large number of papers were published that attempted to address these shortcomings; see Refs. [13-17] for reviews.

### 3.2 Wasserstein Loss with Gradient Penalty

A popular method that has been found to help stabilize GAN training is the Wasserstein loss function used by Arjovsky *et al.* [47]. The Wasserstein GAN (WGAN) aims to minimize the Wasserstein distance between the generated distribution and the target data distribution, where the Wasserstein "earth-mover's" distance can be interpreted as the minimum cost of transporting mass to transform one distribution into another. Due to the intractability of computing the Wasserstein distance directly, Arjovsky *et al.* instead proposed to train the generator to minimize the proxy loss

$L_{W}=\underset{\mathrm{D\epsilon}D}{\max}E_{x∼Pd}[D(x)]-E_{z∼P_{Z}}[D(G(z))],$ (5)

where *D* is the set of 1-Lipshitz functions, and *E*[∙] denotes expected value. When the discriminator is trained to optimality, *i.e.*, the maximum is attained above, then minimizing the value function in Eq. (5) with respect to the generator parameters, *i.e.*, the neural network weights, is equivalent to minimizing the Wasserstein distance between *p_g_* and *p_d_* [47, 48]. Following common practice, the loss function is minimized using the widely used back-propagation algorithm [45, Sec. 6.5]. Arjovsky *et al.* enforced the Lipshitz constraint via weight clipping following every training update.

To further stabilize the WGAN model, Gulranjani *et al.* [48] proposed to add a gradient penalty term to the WGAN loss function instead of weight clipping. The resulting loss, denoted WGAN-GP, minimizes the objective

ℒ$=E_{x∼P_{g}}[D(\tilde{x})]-E_{x∼P_{d}}[D(x)]+\lambda E[{\left(||\nabla_{\hat{X}}D(\hat{x}){\|}_{2}-1\right)}^{2}],$ (6)

where $\hat{x}$ = *εx* + (1 - *ε*)$\tilde{x}$ with $\tilde{x}$ ∼ *p_g_*, *x* ∼ *p_d_*, and *ε* ∼ *U* [0, 1]; *i.e.*, *ε* is drawn from a uniform distribution over the unit interval. Note that $\hat{x}$ is a random linear interpolation between a real data sample, *x*, and a generated data sample, $\tilde{x}$.

Gulrajani *et al.* [48] made several implementation recommendations for WGAN-GP, which we largely followed; see Sec. 6. First, since the WGAN-GP objective penalizes the gradient of the discriminator for each batch independently, the use of batch normalization is not recommended. Second, like Arjovsky *et al.* [47], Gulrajani *et al.* used an imbalanced discriminator-generator update rule, where the discriminator weights were updated five times for each generator update. Third, they recommended using *λ* = 10 for the default gradient penalty weight. Last, Gulrajani *et al.* recommended the ADAM optimizer [49] for discriminator and generator training with default hyperparameter settings *α* = 10^-4^, *β*_1_ = 0, and *β*_2_ = 0.9 for the learning rate and moment decay rates, respectively.

## 4 Synthetic OFDM Data Sets

Later, in Sec. 8, we present four experiments with synthetic OFDM data. This section describes high-level implementation details for our data simulations. Specific parameter settings used in each experiment are given in Sec. 8.

We used synthetic (simulated) data sets of OFDM waveforms, which offer several advantages for early-stage investigations into GAN models. First, since high-quality recordings of real-world OFDM-based communication waveforms are not readily available, and since acquiring such recordings requires significant effort, the ease of creating unlimited amounts of synthetic data is well suited to model development. Second, synthetic data provide control over multiple OFDM parameters, including OFDM symbol length, cyclic prefix, pilot symbols, and resource allocation size, *i.e.*, the number of occupied subcarriers in an OFDM symbol. Last, because real-world communication system recordings involve complicated signaling protocols and suffer from nonideal physical effects, implementing software-based channel equalization and demodulation is difficult. By contrast, synthetic data enable straightforward symbol demodulation and performance evaluation.

Synthetic data sets are constructed by first simulating a different random sequence of bits for each OFDM waveform, where 0 and 1 occur with equal probability. For *M*-ary QAM, each group of *k* = log_2_(*M*) bits is mapped using Gray encoding to QAM symbols [37]. Each block of QAM symbols is then mapped onto a specified collection of OFDM subcarriers, which are modulated into a baseband, I/Q, time-domain waveform by applying an inverse FFT, producing the multicarrier OFDM symbol [37].

Every synthetic waveform consists of a sequence of six OFDM symbols, each with a cyclic prefix equal to 25% of the OFDM symbol length. The OFDM symbol length, *i.e.*, the number of subcarriers, is set to be 128, 256, or 512, yielding full time-series lengths of 960, 1920, or 3840, respectively.^1^1Including the cyclic prefix, each symbol has a length of 160, 320, or 640, respectively. So for a waveform consisting of six symbols, the time series has a length of 960, 1920, or 3840, respectively. The above choices were motivated by the specifications for downlink long-term evolution (LTE) [39, 50, 51]. Namely, symbol lengths of 128, 256, and 512 correspond to LTE channel bandwidths of 1.4 MHz, 3 MHz, and 5 MHz, respectively [51]. The cyclic prefix size corresponds to the so-called "extended" cyclic prefix option in LTE, for which there are six OFDM symbols per 0.5 ms "slot" [50].^2^2For LTE, the physical sampling rate depends channel bandwidth [50].

We considered three different settings for the proportion of occupied OFDM subcarriers, *i.e.*, the resource allocation. Namely, we set the proportion of occupied subcarriers equal to 25%, 50%, or 75% of the maximum allowed for downlink LTE [50, Table 1]. In each case, the block of occupied subcarriers was centered in frequency, and the zero-frequency (DC) subcarrier was not used. For OFDM symbol lengths of 128, 256, and 512, the maximum number of occupied subcarriers, excluding the DC subcarrier, was taken to be 75, 150, and 300, respectively [50, Table 1]. We refer to the three allocation sizes of 25%, 50%, or 75% as small, medium, and large allocations, respectively.

To simulate the effect of thermal noise, additive white Gaussian noise (AWGN) was added to the OFDM signals. In each experiment, the AWGN level was set such that the error vector magnitude (EVM), as defined in Sec. 7, was a specified level.

**Fig. 1:**
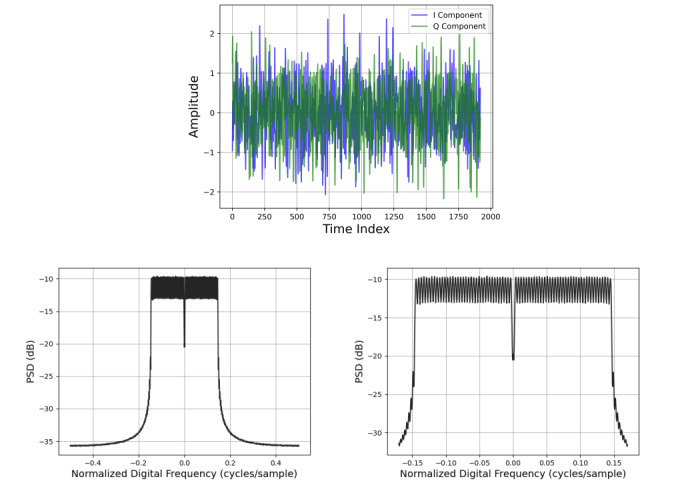
Top: Example synthetic OFDM waveform with symbol length of 256, medium allocation size, and EVM = -25 dB. Lower Left: Estimate of the corresponding power spectral density (PSD). Lower Right: Zoom of PSD focused on occupied subcarriers.

Figure 1 shows an example synthetic OFDM waveform together with a corresponding estimate of the power spectral density (PSD). Here, the OFDM symbol length is 256, 16-QAM is used on each occupied subcarrier, EVM = -25 dB, and the allocation size is medium (50% occupancy). The dip in the PSD at zero frequency arises from the fact that the DC subcarrier is not used.

For our last two experiments in Sec. 8, we performed additional data transformations to simulate physical effects. Namely, the third experiment, presented below in Sec. 8.3, applied 3GPP fading channel models [44, Annex B.2] to the synthetic OFDM data. In addition to application of the random channel model, we modified synthetic data generation for this experiment by inserting a pilot symbol on the 4th OFDM symbol to enable estimation of channel frequency response and equalization coefficients [38]. Here, the pilot symbol was taken to be the first Zadoff-Chu base sequence, as defined for the demodulation reference signal (DMRS) in uplink LTE [39, Sec. 5.5]. Complex-valued Zadoff-Chu sequences are commonly used for channel estimation because they have constant power in time and frequency [52].

The fourth experiment, presented in Sec. 8.4, examined two different types of RF impairments that arise from imperfections in analog RF transmitter and receiver hardware. Namely, we applied models for carrier frequency offset (CFO) and I/Q imbalance; see Smaini [53] for background and details.

CFO originates from a frequency offset in the local oscillator between the transmitter and receiver. CFO in the received baseband signal can be modeled using the relation [53]

*y*(*t*) = *x*(*t*) exp[ *j*2*π*Δ_CFO_*t*], (7)

where *x*(*t*) is the ideal baseband signal, *y*(*t*) is the CFO-impaired baseband signal, and Δ_CFO_ is the CFO spectral shift. To implement this transformation in the discrete-time domain, we set

*y_n_* = *x_n_* exp[ *j*2*π*Δ_CFO_*n/F_s_*], (8)

where Δ_CFO_ is specified in Hertz, *F_s_* is the sampling rate specified in Hertz, and *n* is the time index. 

I/Q imbalance (or mismatch) arises from the fact that quadrature mixers are impaired by gain and phase mismatches. Letting *x_I_*(*t*) and *x_Q_*(*t*) denote the in-phase and quadrature components of the ideal baseband signal, the I/Q imbalance in the transmitter can be modeled as [53, Eq. (2.118)]

*y*(*t*) = $\frac{1-\mathrm{(\Delta}G/2}{2}$ [cos(Δϕ*/*2) + *j*sin(Δϕ*/*2)] *x_I_*(*t*) + $\frac{1+\Delta G/2}{2}$ [sin(Δϕ*/*2) + *j* cos(Δϕ*/*2)] x*_Q_*(*t*), (9)

where *y*(*t*) is the impaired, complex-valued baseband signal, and Δ*_G_* and Δϕ are the gain and phase mismatches between the in-phase (I) and quadrature (Q) branches of the circuit. Here, Δ*_G_* = |*G_I_* - *G_Q_*|*/G_Q_*, where *G_I_* and *G_Q_* are the gains for the I and Q branches, respectively. We applied the above transformation in the discrete-time domain. For simplicity, we only considered I/Q imbalance in the transmitter; a similar model can also be used for I/Q mismatch in the receiver [53].

## 5 Novel Generative Model Architectures

Here, we present two novel GAN models for OFDM signals that build on prior convolutional GANs for time series. Specifically, we propose a direct time-series model and another indirect model based on an image-domain time-frequency representation. Key considerations in our designs included conceptual simplicity and scalability, *i.e.*, the ability of the model to scale to longer and more complex signals, in terms of both computation and performance.

For many practical applications, the OFDM symbol length is known *a priori*; *e.g.*, it is determined by the channel bandwidth for 4G LTE. For this reason, the two models presented below are tailored to OFDM data in the sense that they utilize prior knowledge of the OFDM symbol length. However, all other aspects of the OFDM waveforms, *e.g.*, the QAM symbol constellation, cyclic prefix size, OFDM symbol boundaries, and channel distortions, are assumed to be unknown.

### 5.1 Progressively Scaled Kernel GAN (PSK-GAN)

Our direct time-series model, called progressively scaled kernel GAN (PSK-GAN), uses one dimensional (1-D) convolutional layers and aims to model temporal dynamics by progressively scaling kernel lengths with model depth. Specifically, PSK-GAN employs kernels with lengths progressively scaled up or down by a factor equal to the convolution stride with generator and discriminator model depth, respectively. The motivation behind progressively scaling kernel lengths is to increase the receptive field while avoiding kernels with lengths longer than their inputs. The process of progressively scaling kernel lengths has the effect of scaling the kernel resolution with feature map resolution. The concept of progressively scaling kernel sizes with layer depth was motivated by WaveNet [18], which employs dilated convolutions to achieve a similar outcome.

To avoid the generation of so-called "checkerboard artifacts," which manifest as spikes in the power spectrum, convolutional layer kernel lengths are set to be integer multiples of the stride length, as recommended by Odena *et al.* [54]. Based on empirical testing, we set the maximum kernel length equal to the OFDM symbol length and the minimum allowable kernel length to 4.

Tables 1 and 2 outline the PSK-GAN architectures for the generator and discriminator, respectively. In these tables, Dense, Conv 1-D and Transpose Conv 1-D, denote dense fully connected layers, one-dimensional convolutional layers, and transposed convolutional layers, respectively. Also, Tanh, ReLU, and LReLU indicate hyperbolic-tangent (Tanh), rectified linear unit (ReLU), and leaky rectified linear unit (LReLU) activation functions. The filter dimensions for convolutional layers correspond to kernel length, number of input channels, and number of output channels, respectively. Here, *f* = 1, 2, or 4 for OFDM symbol sizes of 128, 256, and 512, respectively, and *n* is the batch size. Similarly, the filter dimensions for the dense layers correspond to input length and output length respectively. To yield time-series lengths that are compatible with convolutional layers with strides of 4, target OFDM signals are zero-padded to the nearest power of 2.

**Table 1:** PSK-GAN generator architecture [ *f* = 1, 2, 4].

**Operation**	**Filter Shape**	**Output Shape**
*z* ∼ Uniform(-1, 1)		(*n*, 100)
Dense	(100, 1024 *f*)	(*n*, 1024 *f*)
Reshape		(*n*, 1024, *f*)
ReLU		(*n*, 1024, *f*)
Transpose Conv1-D (stride=4)	(4 *f*, 1024, 512)	(*n*, 512, 4 *f*)
ReLU		(*n*, 512, 4 *f*)
Transpose Conv1-D (stride=4)	(4 *f*, 512, 256)	(*n*, 256, 16 *f*)
ReLU		(*n*, 256, 16 *f*)
Transpose Conv1-D (stride=4)	(8 *f*, 256, 128)	(*n*, 128, 64 *f*)
ReLU		(*n*, 128, 64 *f*)
Transpose Conv1-D (stride=4)	(32 *f*, 128, 64)	(*n*, 64, 256 *f*)
ReLU	(128 *f*, 64, 2)	(*n*, 64, 256 *f*)
Transpose Conv1-D (stride=4)	(128 *f*, 64, 2)	(*n*, 2, 1024 *f*)
Tanh		(*n*, 2, 1024 *f*)

**Table 2:** PSK-GAN discriminator architecture [ *f* = 1, 2, 4].

**Operation**	**Filter Shape**	**Output Shape**
*x* ∼ *G*(*z*)		(*n*, 2, 1024 *f*)
Conv1-D (stride=4) LReLU(*α* = 0.2)	(128 *f*, 2, 64)	(*n*, 64, 256 *f*) (*n*, 64, 256 *f*)
Conv1-D (stride=4) LReLU(*α* = 0.2)	(32 *f*, 64, 128)	(*n*, 128, 64 *f*) (*n*, 128, 64 *f*)
Conv1-D (stride=4) LReLU(*α* = 0.2)	(8 *f*, 128, 256)	(*n*, 256, 16 *f*) (*n*, 256, 16 *f*)
Conv1-D (stride=4) LReLU(*α* = 0.2)	(4 *f*, 256, 512)	(*n*, 512, 4 *f*) (*n*, 512, 4 *f*)
Conv1-D (stride=4) LReLU(*α* = 0.2)	(4 *f*, 512, 1024)	(*n*, 1024, *f*) (*n*, 1024, *f*)
Reshape		(*n*, 1024 *f*)
Dense	(1024 *f*, 1)	(*n*, 1)

### 5.2 Short-Time Fourier Transform GAN (STFT-GAN)

As mentioned in Sec. 1, many time-series GANs train the generator to produce an image-domain time-frequency representation that is then mapped into a time series. Motivated by these approaches, we propose a two-dimensional (2-D) convolutional model, called short-time Fourier transform GAN (STFT-GAN), that is trained on a complex-valued short-time Fourier transform (STFT) representation of the OFDM time series. Similar GANs based on STFT representations have also been used for audio generation, *e.g.*, Refs. [23, 25]. Our model differs from these prior works in two ways. Namely, our model uses different network architectures and it directly uses the complex-valued STFT without additional processing. Related approaches that apply additional processing to the STFT, *e.g.*, those in Refs. [23, 25], were not found to be advantageous in preliminary tests with synthetic OFDM data sets.

The STFT (a.k.a. windowed Fourier transform) is computed by dividing the time series into overlapping segments of equal length, applying a window function, and then calculating the DFT on each segment [55, 56]. We used a Hann window and 75% segment overlap. The Hann window was used since it is a common default choice, and the amount of overlap was selected to be consistent with methods in Refs. [23, 25]. Under these conditions, the constant-overlap-add (COLA) constraint is satisfied [55], and the STFT is invertible; *i.e.*, no information is lost.^3^3Noninvertible STFTs were not considered, since they do not ensure that samples of the generated distribution have the same dimensionality as samples of the target distribution.

OFDM target waveforms are first zero-padded to the nearest power of 2 before conversion to an STFT representation. We set the STFT window length equal to the OFDM symbol length, since empirical tests indicated that this choice gives superior results. The STFT values are rescaled to the range [-1, 1] and shifted such that the zero-frequency component is at the center of each DFT window.

The architecture of STFT-GAN is based on the DCGAN architecture [28], with modifications made to accommodate the nonsquare shape of the STFT. Specifically, STFT-GAN is composed of four 2-D convolutional layers with 4 × 4 kernels for both the generator and discriminator; see Tables 3 and 4. In the tables, the notation is similar to that used for PSK-GAN, with Conv 2-D and Transpose Conv 2-D indicating two-dimensional convolutional and transposed convolutional layers, *f* = 1, 2, or 4 corresponding to waveforms with OFDM symbol lengths of 128, 256, and 512, respectively, and *n* denoting the batch size.

**Table 3:** STFT-GAN generator architecture [ *f* = 1, 2, 4].

**Operation**	**Filter Shape**	**Output Shape**
*z* ∼ Uniform(-1, 1)		(*n*, 100)
Dense	(100, 16384 *f*)	(*n*, 16384 *f*)
Reshape		(*n*, 1024, 8 *f*, 2)
ReLU		(*n*, 1024, 8 *f*, 2)
Transpose Conv2-D (stride=2) ReLU	(4, 4, 1024, 512)	(*n*, 512, 16 *f*, 4) (*n*, 512, 16 *f*, 4)
Transpose Conv2-D (stride=2)	(4, 4, 512, 256)	(*n*, 256, 32 *f*, 8)
ReLU		(*n*, 256, 32 *f*, 8)
Transpose Conv2-D (stride=2)	(4, 4, 256, 128)	(*n*, 128, 64 *f*, 16)
ReLU		(*n*, 128, 64 *f*, 16)
Transpose Conv2-D (stride=2) Tanh	(4, 4, 128, 2)	(*n*, 2, 128 *f*, 33) (*n*, 2, 128 *f*, 33)

**Table 4:** STFT-GAN discriminator architecture [ *f* = 1, 2, 4].

**Operation**	**Filter Size**	**Output Shape**
*x* ∼ *G*(*z*)		(*n*, 2, 128 *f*, 33)
Conv2-D (stride=2) LReLU(*α* = 0.2)	(4, 4, 2, 128)	(*n*, 128, 64 *f*, 16) (*n*, 128, 64 *f*, 16)
Conv2-D (stride=2) LReLU(*α* = 0.2)	(4, 4, 128, 256)	(*n*, 256, 32 *f*, 8) (*n*, 256, 32 *f*, 8)
Conv2-D (stride=2) LReLU(*α* = 0.2)	(4, 4, 256, 512)	(*n*, 512, 16 *f*, 4) (*n*, 512, 16 *f*, 4)
Conv2-D (stride=2) LReLU(*α* = 0.2)	(4, 4, 512, 1024)	(*n*, 512, 8 *f*, 2) (*n*, 512, 8 *f*, 2)
Reshape		(*n*, 16384 *f*)
Dense	(16384 *f*, 1)	(*n*, 1)

## 6 Training Protocol

Both PSK-GAN and STFT-GAN were trained with WGAN-GP loss described in Sec. 3.2. Like the WGAN-GP training protocol, PSK-GAN and STFT-GAN were trained using the ADAM optimizer [49] for discriminator and generator with hyperparameter settings of *α* = 10^-4^, *β*_1_ = 0, and *β*_2_ = 0.9 for the learning rate and moment decay rates, respectively. In a departure from the WGAN-GP protocol, we used a 1:1 update ratio between the discriminator and generator, modified from the original 5:1 ratio. This choice was found to yield better convergence on our target data sets. We trained each model with a target data set of size 2^16^ = 65536, for 500 epochs with a batch size of 128.

Following common practice with GANs, the training data were scaled to the range [-1, 1], which corresponds to the range of the tanh output activation of the generator [28]. Specifically, for STFT-GAN, all target distributions were scaled using feature-based min-max scaling, which scales the minimum and maximum values of each time step to [-1, 1]. By contrast, for PSK-GAN, the target distribution was scaled with min-max scaling using the global minimum and maximum values. Global min-max scaling was used for PSK-GAN since it was found to yield better results. All outputs from the generator were rescaled back to the original range using the applicable inverse transformation.

## 7 Evaluation Methods

Evaluations of GANs often focus on subjective assessments of perceptual quality or quantitative metrics that require a suitable feature space defined by, for example, a pretrained model on a standard data set [57, 58]. Since OFDM waveforms are not directly human interpretable (*e.g.*, see Fig. 1), it is not possible to assess OFDM signal fidelity on a perceptual basis. Moreover, there are no standard pretrained classification models for time series, so general-purpose quantitative GAN evaluation measures that require a suitable feature space are not easily applied. Therefore, we focused our evaluations on OFDM-specific signal attributes. Namely, we evaluated the quality of the PSD, the QAM constellation, and the cyclic prefix.

All evaluations were conducted with test sets that were 1*/*4 the size of the training set. Test sets had size of 2^14^ = 16384. Test sets of target waveforms were created independently of the training set, and test sets of generated waveforms were created at the completion of training.

To avoid parametric assumptions, we estimated the PSD by applying the multitaper method [59, 60], a versatile nonparametric approach, to the full duration of each waveform. The number of frequency bins was taken as the next power of 2 greater than or equal to the waveform length. To obtain a representative PSD estimate across the test set, we took the median value in each frequency bin; see Fig. 1 for an example median PSD estimate.

Let the median PSDs for the target and generated distributions be denoted as *P_t_*(*f_d_*) and *P_g_*(*f_d_*), respectively, where *f_d_* ∈ [-0.5, 0.5] is normalized digital frequency with units of cycles per sample. To assess the accuracy of *P_g_* relative to *P_t_*, we used the "geodesic distance" for power spectra proposed by Georgiou [61, 62], defined as

$d_{g}(P_{g},P_{t})=\sqrt{{\int_{-0.5}^{0.5}\left(\log\frac{P_{g}(f_{d})}{P_{t}(f_{d})}\right)}^{2}df_{d}-{\left(\int_{-0.5}^{0.5}\log\frac{P_{g}(f_{d})}{P_{t}(f_{d})}df_{d}\right)}^{2}}.$

(10)

The above quantity can be interpreted as the length of a geodesic connecting points on the manifold of PSDs [61]. Notably, this distance does not distinguish PSDs that differ by a constant, positive, multiplicative factor [62]. Also, note that the first term is equivalent to a difference of log-transformed power spectra, capturing PSD differences across a large dynamic range.

Denoting the discrete-valued PSDs as *P_g_*[*k*] and *P_t_*[*k*], and approximating the integrals with summations, we obtain the discrete form

$d_{g}(P_{g},P_{t})\approx \sqrt{{\sum_{k}\left(\log\frac{P_{g}[k]}{P_{t}[k]}\right)}^{2}\Delta f_{d}-{\left(\sum_{l}\log\frac{P_{g}\left[l\right]}{P_{t}\left[l\right]}\Delta f_{d}\right)}^{2}},$ (11)

where the summations are taken over the index for the normalized digital frequency grid with step size Δ *f_d_*. Since the choice of logarithm above is arbitrary, we chose to implement the above formula with a natural logarithm.

To quantitatively evaluate the quality of the QAM constellation, we used EVM, which measures the root mean square (RMS) deviation of measured symbols from the ideal signal constellation [63, 64]. Namely, we used the commonly used definition [63]

$\text{EVM=}\sqrt{\frac{\frac{1}{N_{S}}\sum_{i=1}^{N_{S}}|S_{\text{meas},i}-S_{\text{ideal},i}{|}^{2}}{\frac{1}{M}\sum_{i=1}^{M}|S_{\text{ideal},i}{|}^{2}},}$

where *N_s_* is the number of symbols in a random symbol sequence, *M* is the number of unique symbols in the constellation, *S*_meas_*_,i_* is the *i*th measured symbol, and *S*_ideal_*_,i_* is the ideal constellation point for the *i*th symbol. Above, it is assumed that the number of symbols in the sample, *N_s_*, is large enough to ensure that all possible symbols and transitions are observed [63]. To define added noise levels and to assess signal fidelity, we used the above definition of EVM expressed in decibels (dB), *i.e.*, 20 log_10_ EVM. We estimated EVM for a single waveform using the set of all QAM symbols in the waveform and then found the median EVM value across all waveforms in a test set.

**Fig. 2:**
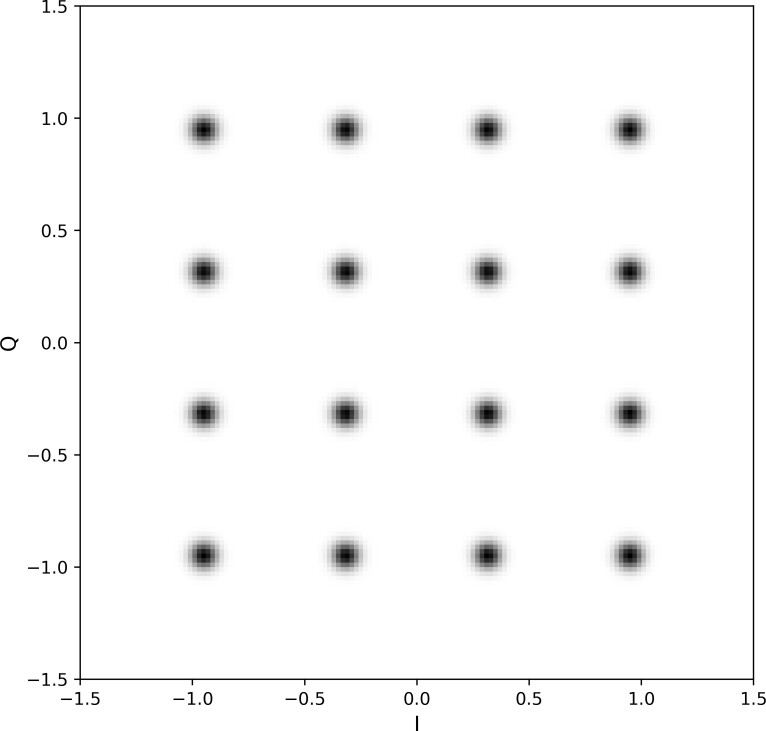
Example constellation diagram for a 16-QAM signal constellation with EVM = -25 dB.

We supplemented EVM assessment with qualitative evaluation of constellation diagrams, a commonly used method to visualize digitally modulated signals. A conventional constellation diagram is a scatter plot in I/Q space of the sequence of measured symbols. Since the total number of QAM symbols in the test set was very large, we plotted 2-D histograms instead of scatter plots. Specifically, we plotted constellation diagrams using 2-D histograms with 150 × 150 bins, evenly spaced over the region [-1.5, 1.5] × [-1.5, 1.5]. An example 16-QAM constellation diagram for a test set with EVM = -25 dB is shown in Fig. 2.

The presence and quality of a cyclic prefix at the beginning of each OFDM symbol were evaluated as follows. First, for each waveform in the target and generated test sets, we found the cross-correlation function of each cyclic prefix with the waveform, where all cyclic prefix segments were removed. The location and strength of the cross-correlation maximum indicated the accuracy of the cyclic prefix in the generated waveforms. We obtained an aggregate metric for cross-correlation strength by finding the median of the maximum cross-correlation values across the generated and target test sets, denoted R_gen_ and R_target_ respectively, and then computing the relative error, expressed as a percentage,

${\text{RelErr}}_{\text{CP}}\%=\frac{{\text{|R}}_{\text{gen}}{\text{-R}}_{\text{target}}|}{\left|{\text{R}}_{\text{target}}\right|}\times 100$. (13)

As mentioned earlier in Sec. 4, our third experiment, presented in Sec. 8.3, applied fading channel models to the synthetic OFDM data. To extract QAM symbols and evaluate the signal constellation, we first equalized the OFDM data on each subcarrier to correct for channel effects, a standard step prior to decoding received OFDM signals. Namely, the channel frequency response for each block of 6 OFDM symbols was estimated across occupied subcarriers by taking the demodulated pilot symbol located at the 4th OFDM symbol location and dividing it by the known pilot sequence. The QAM symbol on each subcarrier was then equalized by dividing by the corresponding estimated channel frequency response coefficient [38].

A metric commonly used to characterize channels with frequency-selective fading is the coherence bandwidth, defined as the half width at half maximum of the channel's time-frequency correlation function [38]. Because the coherence bandwidth characterizes the correlations between fades at different frequencies, it is particularly relevant for schemes like OFDM that transmit on multiple subcarriers [38]. For this reason, we used coherence bandwidth as a performance metric when we investigated fading channels in the experiment in Sec. 8.3.

To estimate coherence bandwidth, we computed the autocorrelation function of the channel frequency response estimated with each pilot symbol and found the half width at half maximum. We then plotted histograms of the estimated values across the whole test set.

## 8 Experiments

Below, we present the results of four experiments: a data complexity experiment, a modulation-order experiment, a fading channel experiment, and an RF impairment experiment.

### 8.1 Data Complexity Experiment

The objective of the data complexity experiment was to evaluate how well our GAN models performed as the target OFDM data set became increasingly complex. Namely, we changed two experimental factors: OFDM symbol length and the resource allocation size (proportion of occupied subcarriers). For details on how settings for these factors are implemented, see Sec. 4. We used three settings for the OFDM symbol length, 128, 256, and 512, and three settings for the allocation size, denoted small, medium, and large, resulting in a total of nine test configurations. All target data sets used 16-QAM digital modulation on the occupied OFDM subcarriers, and AWGN was added such that EVM = -25 dB. This EVM value was selected because it corresponds to a noticeable level of noise that results in essentially no bit errors, *i.e.*, a strong communication link [65]. We compared the two GAN models presented in Sec. 5, PSK-GAN and STFT-GAN, to an implementation of WaveGAN [20], described in the Appendix. WaveGAN was chosen as a baseline model for comparison because it is a state-of-the art direct time-series GAN.

To assess training variability, all models were trained on each data set three times, with different neural network weight initialization and different batch randomization. On our computational hardware, the training time for each model was approximately 12 h. Therefore, due to the high computational cost associated with training multiple model instances, it was necessary to limit the number of repetitions. For this reason, three repetitions of each configuration was selected to gain limited insights into training variability.

**Fig. 3:**
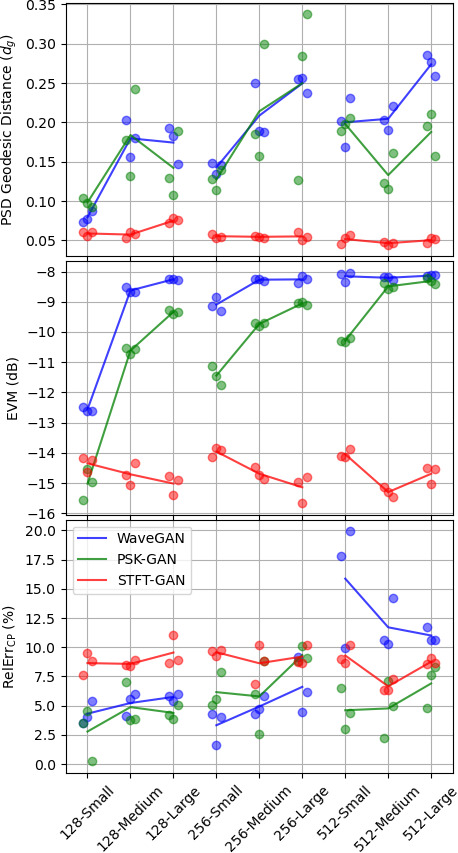
Aggregate results for the data complexity experiment. Top: Power spectral density (PSD) geodesic distance. Middle: Error vector magnitude (EVM). Bottom: Relative error in maximum cyclic prefix cross-correlation.

Figure 3 summarizes the results of the data complexity experiment for the PSD, EVM, and cyclic prefix evaluations, respectively. The *x*-axis tick labels indicate the OFDM symbol length and the allocation size; *e.g.*, "128-Small" denotes the test configuration with a 128 symbol length and small allocation size. In these plots, the results for each model repetition are shown with circles. Also, lines connecting average values across the three repetitions are shown to aid visual interpretation. Error bars were omitted from the plots since the dominant source of uncertainty was training variability as reflected by the spread of the three repetitions, and the uncertainties for individual model results were too small to be visible.

The PSD results in Fig. 3 (top) show that STFT-GAN consistently had the smallest PSD distance relative to the target distribution and was fairly consistent across test conditions. On the other hand, PSK-GAN and WaveGAN displayed a wider range of performance, with much larger PSD distances in many cases.

**Fig. 4:**
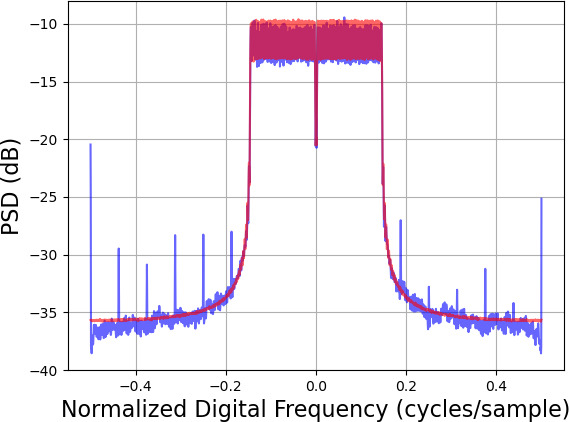

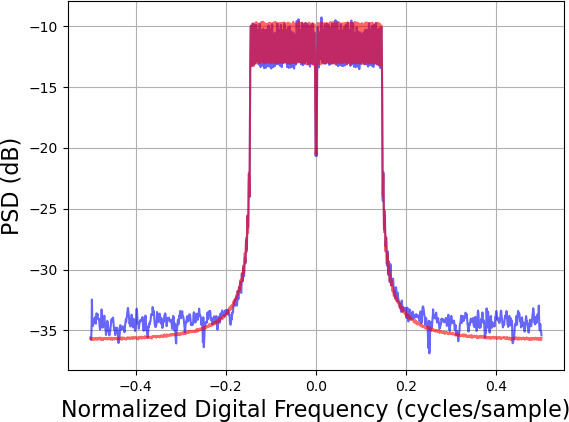

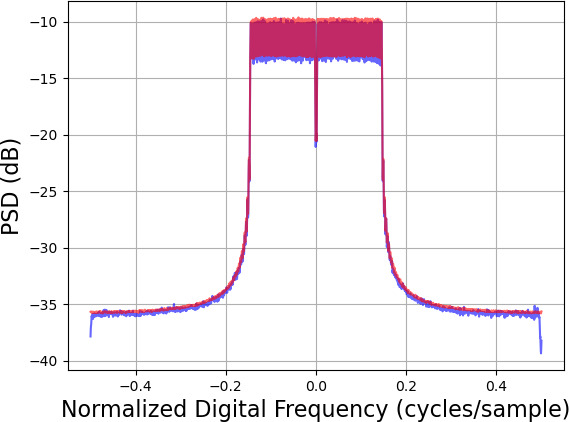
Estimated median power spectral densities (PSDs) for the data complexity experiment with 256 OFDM symbol length and medium allocation size. The PSD for the target distribution is shown in red, and the PSD for the generated distribution is shown in blue. Left: WaveGAN. Middle: PSK-GAN. Right: STFT-GAN.

Estimated median PSDs for the 256-Medium condition are shown in Fig. 4. These plots show that the PSD for the generated distribution from WaveGAN suffered from spikes, likely due to the so-called "checkerboard artifact" phenomenon; see the Appendix. Also, the spectral density for PSK-GAN is seen to be higher than the target distribution in areas without occupied subcarriers. On the other hand, the PSD for STFT-GAN shows excellent agreement with the target distribution.

The EVM results in Fig. 3 (middle) display clear trends in performance. Specifically, the direct time-series models, PSK-GAN and WaveGAN, showed worsening performance as both the OFDM symbol size and allocation size increased, with PSK-GAN edging out WaveGAN, especially for small allocation sizes. By contrast, STFT-GAN markedly outperformed the direct time-series models and did not display a degradation in performance as the symbol size and allocation size increased. The EVM achieved by STFT-GAN ranged between -14 dB and -16 dB, indicating that the recovered constellation fidelity was worse than the target EVM of -25 dB by as much as 9 dB. Clues to the source of the lower fidelity are visible in the constellation diagrams described next.

**Fig. 5:**
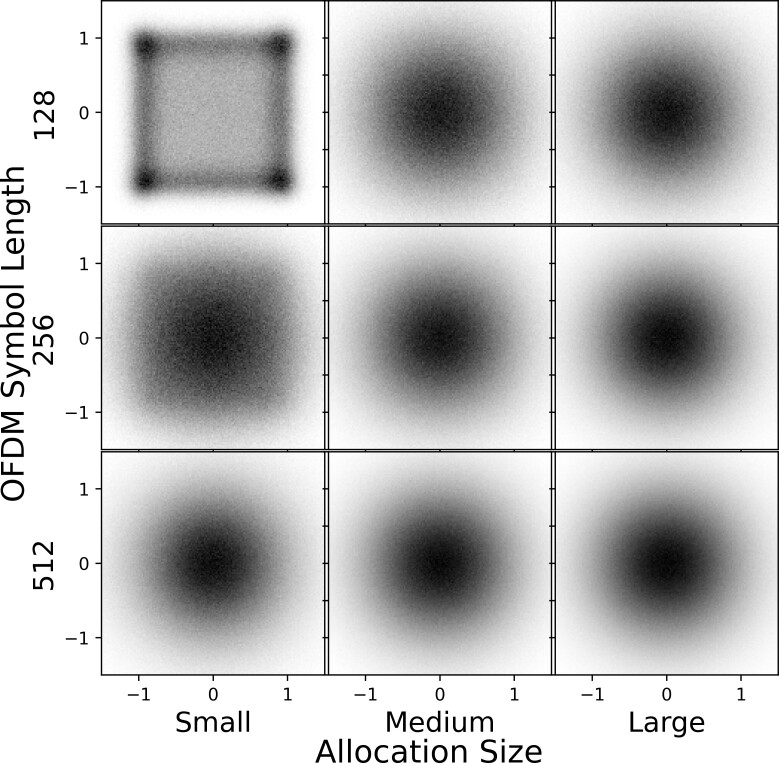

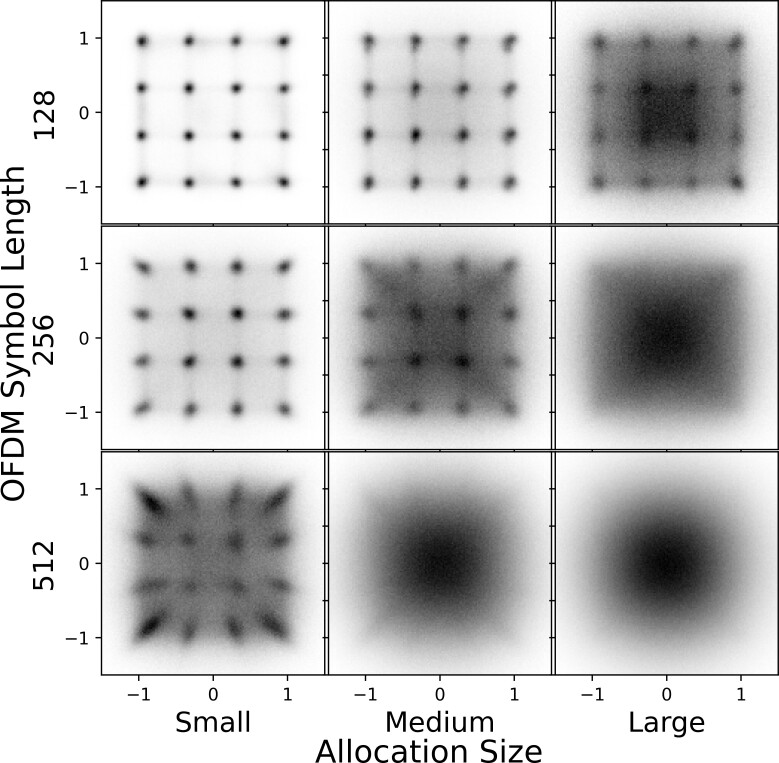

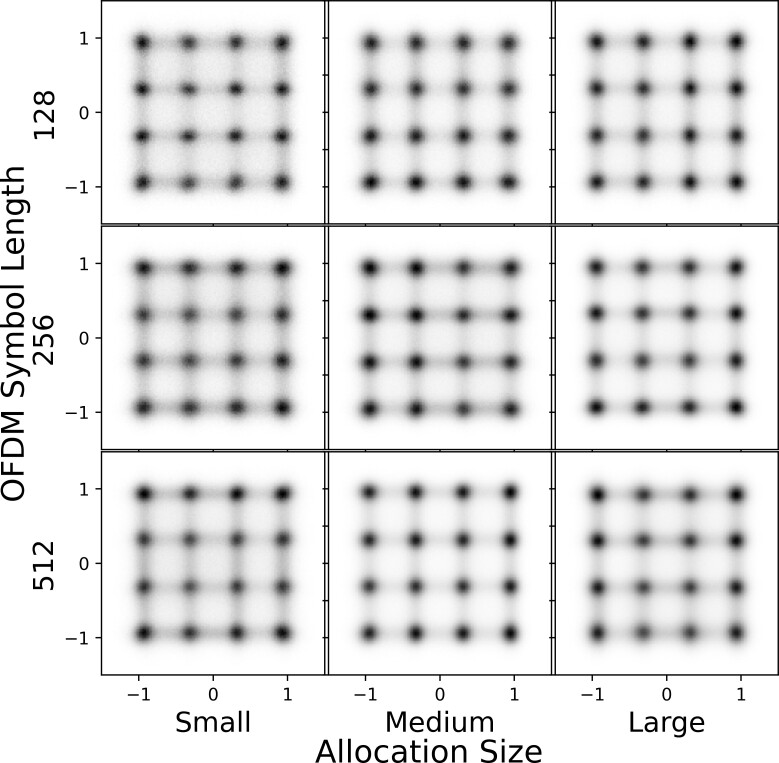
Constellation diagrams for the data complexity experiment. Left: WaveGAN. Middle: PSK-GAN. Right: STFT-GAN.

Figure 5 displays constellation diagrams for the three GAN models; compare these diagrams to Fig. 2 for the target distribution. The plots in Fig. 5 show clear qualitative differences in performance between the models. Specifically, for all conditions, WaveGAN struggled to learn the full 16-QAM constellation.

PSK-GAN successfully learned the 16-QAM constellation in the simpler conditions with smaller allocations and shorter symbol lengths, but it performed worse as the symbol length and allocation size increased. On

**Fig. 6:**
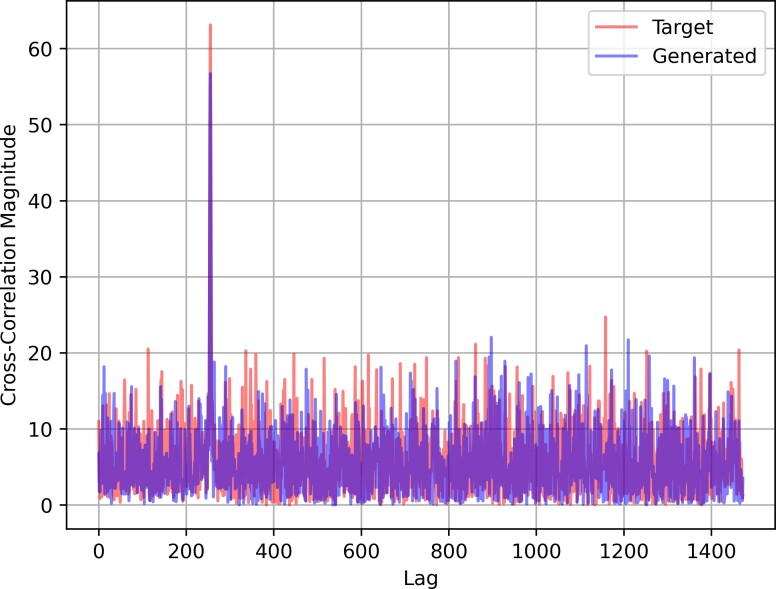
Median cross-correlation magnitude of all cyclic prefixes and OFDM symbols in the 256-medium condition for STFT-GAN.

the other hand, STFT-GAN clearly learned the 16-QAM symbol constellation under all conditions. However, as noted above for the EVM results, the observed constellation fidelity for STFT-GAN did not match the target distribution. For example, there are visible grid lines between symbols that can be interpreted as a form of mode mixing.

The cyclic prefix results are shown in Fig. 3 (bottom). WaveGAN performed fairly well for the 128 and 256 symbol lengths, but performance was much worse for the 512 symbol length, with relative errors above 10%. On the other hand, PSK-GAN and STFT-GAN had relatively uniform performance across all conditions, with PSK-GAN displaying slightly better relative errors below 10%.

Figure 6 shows the median cross-correlation of all cyclic prefixes and OFDM symbols for generated and target distributions, respectively, in the 256-medium condition for STFT-GAN. Here, each cyclic prefix is cross-correlated with the whole waveform with the cyclic prefixes removed. The lags are normalized so that zero lag corresponds to the first time step of the OFDM symbol associated with each cyclic prefix. The plot for the generated waveform distribution shows a peak correlation in the expected location at the end of each OFDM symbol. Also, the cross-correlation plot indicates insignificant correlation with the remaining OFDM symbols. These results are representative of the findings for all other conditions and models; *i.e.*, all models learned the cyclic prefix location accurately.

Overall, based on the superior performance of STFT-GAN as evidenced by the PSD and EVM metrics, as well as the constellation diagrams, we conclude that STFT-GAN is the most effective of the three models at adapting to increasing OFDM data set complexity. Note that all three models had a similar number of weights and, hence, comparable computational requirements.

We speculate that the better performance of STFT-GAN may be due to the fact that the STFT representation was calculated with an STFT window size equal to the OFDM symbol length, which implies that each pixel resolves a single subcarrier. On the other hand, the direct waveform models use a data representation in which all subcarriers are superimposed in time. Due to the superiority of STFT-GAN, the following experiments focused specifically on STFT-GAN.

### 8.2 Modulation-Order Experiment

The goal of the modulation-order experiment was to assess how STFT-GAN performed with different QAM modulation orders, which are dynamically adjusted in LTE and WLANs to adapt the data transmission rate to the propagation channel [39, 40]. We considered modulation orders of *M* = 4, 16, 32, and 64, respectively. In all cases, the OFDM symbol length was set to 128, the allocation size was medium, and the target EVM was -25 dB.

**Fig. 7:**
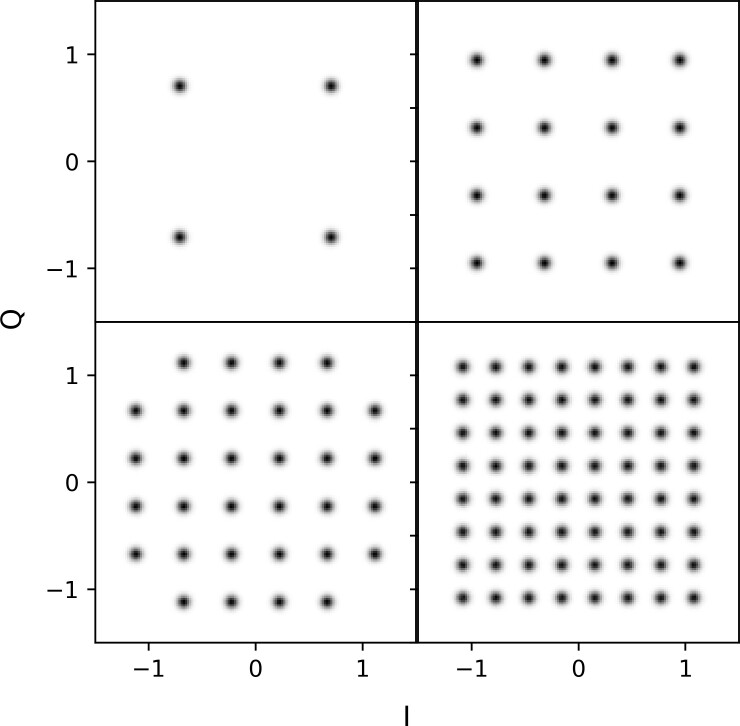
Results for the modulation-order experiment. Left: Target distribution constellations for 4-QAM (upper left), 16-QAM (upper right), 32-QAM (lower left), and 64-QAM (lower right). Right: Generated distribution constellations.

Figure 7 shows constellation diagrams for the four modulation orders. Performance worsened with increasing modulation order, with the constellation not clearly recovered for *M* = 32 and *M* = 64. This result is expected, since increasing the modulation order both increases the total number of symbols and decreases the distance between symbols in I/Q space. For all cases, the PSD distances ranged between 0.05 and 0.075, and the EVM values for all cases were between -17 dB and -14.5 dB, both of which were consistent with the findings in the data complexity experiment.

### 8.3 Fading Channel Experiment

The objective of the fading channel experiment was to evaluate the ability of STFT-GAN to learn waveform variations due to the frequency-selective fading that arises from multipath RF propagation. Specifically, we applied stochastic *N*-tap Rayleigh fading channel models [38] to the target distribution OFDM waveforms. We used three channel models specified in the 3GPP cellular standard [44, Annex B.2]: EPA-5 Hz, EVA-70 Hz, and ETU-300 Hz. The first three letters specify a delay profile, and the frequency is the maximum Doppler frequency.

To be sensitive to frequency-dependent channel variations, the largest OFDM symbol length of 512 was used. In addition, the allocation size was medium, and the QAM modulation order was *M* = 16. To partially offset the nonuniform level of distortion from the different channel models, AWGN was added such that the EVM was -30 dB, -40 dB, and -50 dB before application of the EPA-5 Hz, EVA-70 Hz, and ETU-300 Hz channel models, respectively.

Because the 3GPP channel models are specified in terms of physical quantities, their implementation requires specification of a physical sampling rate. We used a sampling rate of 7.68 megasamples per second, which corresponds to the 3GPP specification for a 5 MHz LTE downlink channel, where the OFDM symbol length is 512 [50].

**Fig. 8:**
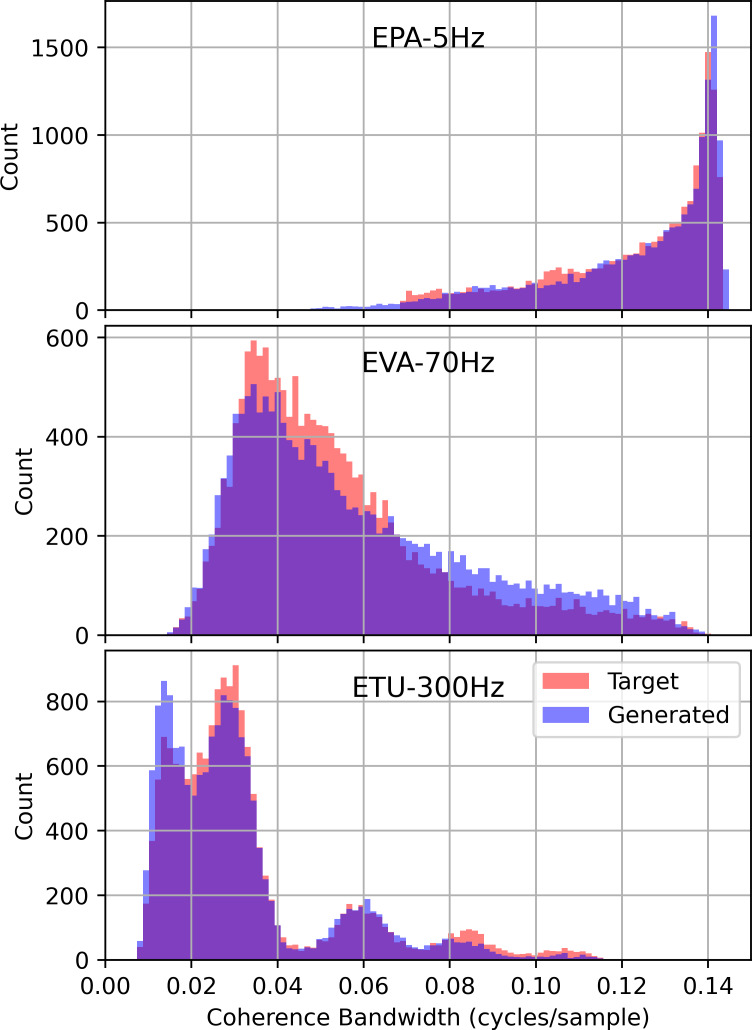
Histograms of estimated coherence bandwidth for the fading channel experiment.

Figure 8 presents histograms of estimated coherence bandwidth for the three channels. In each case, these plots show excellent agreement between the distributions of estimated coherence bandwidth for the generated and target data distributions. Moreover, for all cases, the PSD distances ranged between 0.075 to 0.1, indicating strong median PSD accuracy.

After channel equalization, the EVMs of the target and generated distributions were -26.8 dB and -12.1 dB, -18.1 dB and -11.3 dB, and -7.8 dB and -10.1 dB, for the EPA-5 Hz, EVA-70 Hz, and ETU-300 Hz channel models, respectively. These results are consistent with the findings of the data complexity experiment, indicating that there is a limit to the constellation fidelity for lower target EVMs. Thus, while STFT-GAN failed to achieve target constellation fidelity, it successfully learned the target PSD and channel effects quantified by the coherence bandwidth.

### 8.4 RF Impairment Experiment

The aim of the RF impairment experiment was to assess the ability of STFT-GAN to learn effects arising from imperfections in analog RF hardware. Namely, we examined two types of RF impairments, carrier frequency offset (CFO) and I/Q imbalance; see Sec. 4 for implementation details. To better understand the impact of each impairment, we conducted separate subexperiments on CFO and I/Q imbalance, respectively. For all test configurations, we used an OFDM symbol length of 256, a QAM modulation order of *M* = 16, and the large allocation size. Additionally, we added AWGN such that the EVM = -25 dB prior to application of the impairment transformation.

For the CFO subexperiment, relative to a physical sampling rate of 3.84 megasamples per second, which corresponds to the 3GPP specification for a 3 MHz LTE downlink channel where the OFDM symbol length is 256 [50], we considered two settings: Δ_CFO_ = 50 Hz and 300 Hz. Since the subcarrier spacing in LTE is 15 kHz, these CFO values correspond to 0.33% and 2% of the subcarrier spacing, respectively. For the I/Q imbalance subexperiment, we considered two configurations: (Δ *_G_*, Δϕ) = (10%, 5°) and (20%, 10°), which we denote as "low" and "high" I/Q imbalance conditions, respectively.

Because the CFO and I/Q impairments had minimal effects on the PSD, we do not present PSD results here. In all cases, the PSDs for generated distributions showed strong agreement with the target distributions, similar to those observed previously.

**Fig. 9:**
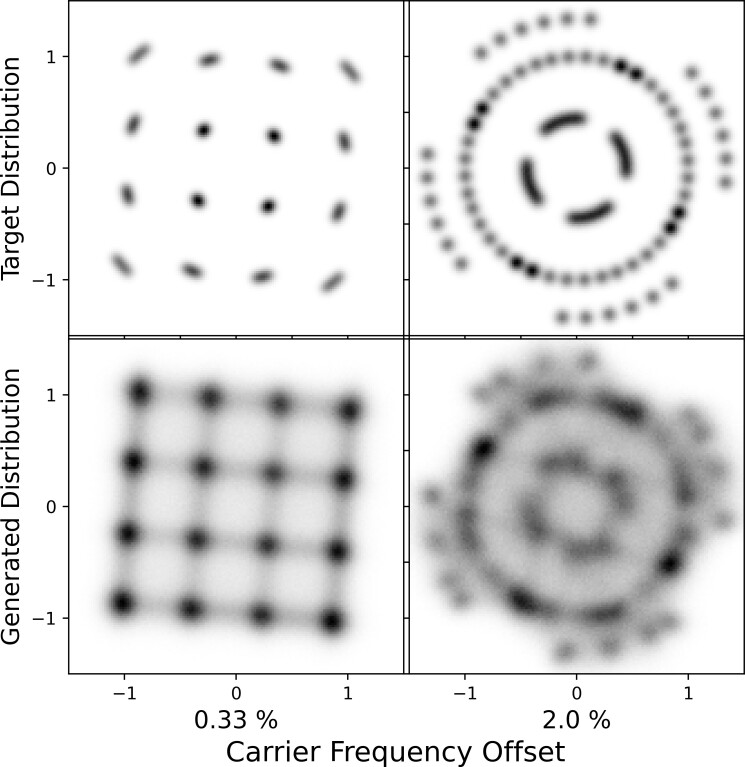

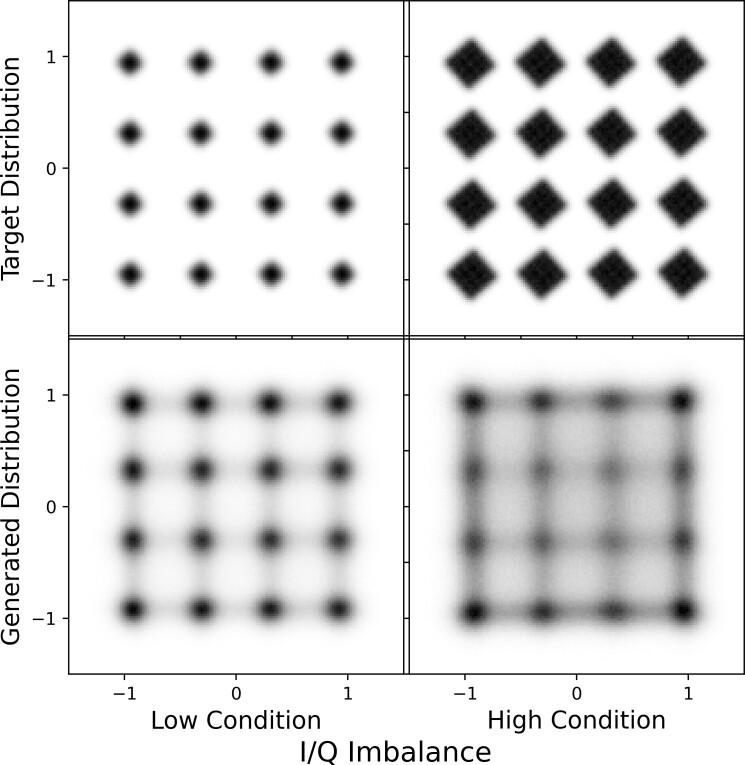
Results for the RF impairment experiment. Left: Constellation diagrams for the carrier frequency offset (CFO) subexperiment; the CFO percentages denote frequency offset relative to subcarrier spacing. Right: Constellation diagrams for the I/Q imbalance subexperiment.

Figure 9 presents constellation diagrams for the RF impairment experiment. The top row shows the target distributions, and the bottom row shows the generated distributions. The left plot, which shows the CFO experimental results, demonstrates that STFT-GAN successfully learned several aspects of the CFO impairment, such as the symbol locations and constellation rotation. On the other hand, the overall fidelity of the STFT-GAN results clearly did not match the target distributions, which is consistent with the findings of the prior experiments. The right plot, which contains the I/Q imbalance experimental results, shows that STFT-GAN did not produce the effects caused by I/Q imbalance on the constellation. Considering the previously observed fidelity limitations in generated constellations, this finding is not surprising, since the I/Q mismatch induces relatively subtle changes in the constellation that are below the precision of the generator.

## 9 Discussion and Conclusions

Building on prior GAN methods, we proposed two novel GAN models, PSK-GAN and STFT-GAN, for I/Q OFDM time series and evaluated their performance, along with a previously published model, WaveGAN, using simulated data sets with known ground truth. Specifically, we investigated model performance with respect to increasing data set complexity over a range of OFDM parameters and conditions, including fading channels and RF impairments. In all cases, performance evaluations were focused on metrics specific to communication signals, such as PSD and signal constellation fidelity.

In the data complexity experiment, we found that a GAN based on an image-domain time-frequency representation, STFT-GAN, demonstrated superior performance to two GANs that directly modeled time series, PSK-GAN and WaveGAN. Namely, STFT-GAN had better PSD fidelity while maintaining lower EVM across increasingly complex OFDM scenarios. By contrast, both direct time-series models did not perform as well when the OFDM symbol length and allocation size increased. Because both direct time-series models had worsening performance for longer OFDM symbol lengths and larger proportions of occupied subcarriers, they were not found to be viable candidates for further study. Hence, the remaining two experiments focused exclusively on STFT-GAN.

Perhaps not surprisingly, the modulation-order experiment demonstrated that increasing the QAM modulation order with the same target EVM resulted in a decreased ability to learn the symbol constellation. Specifically, with a target EVM = -25 dB, STFT-GAN did not learn the *M* = 64 and *M* = 32 constellations as well as the *M* = 16 and *M* = 4 constellations. This observation is likely due to the fact that when the signal power is held constant, the density of constellation symbols in I/Q space increases with modulation order. Future studies with higher-order modulation schemes may need to overcome this limitation.

The fading channel experiment showed that STFT-GAN accurately learned the expected distribution of estimated coherence bandwidth for each channel type. Moreover, STFT-GAN learned generated distributions with median PSDs that closely matched the target distributions. Thus, this experiment demonstrated that GANs are capable of learning signal distortions due to stochastic fading channels.

The RF impairment experiment found that STFT-GAN was able to successfully produce several aspects of the CFO-impaired signal constellation. By contrast, STFT-GAN did not produce the effects caused by I/Q imbalance on the constellation. For both impairments, it was clear that the precision realized by STFT-GAN was insufficient to fully capture these subtle effects on the signal constellation.

In all experiments, STFT-GAN achieved excellent PSD accuracy, including in regions outside the set of active subcarriers. Moreover, it reliably learned the time-domain cyclic prefix location. On the other hand, while STFT-GAN learned the QAM signal constellation in many cases, it did not achieve the desired constellation fidelity as measured by EVM. This finding indicates a limitation of the STFT-GAN model and training protocol employed in this work. The potential for additional model optimization, *e.g.*, deeper models and longer training, to improve constellation fidelity could be explored in future studies.

Overall, these findings indicate those use cases that are feasible for STFT-GAN as well as those where additional advances are needed. Namely, STFT-GAN is likely not a good candidate for end-to-end communication system modeling, unsupervised encoding/decoding, or bit-error-rate testing where it is important to achieve accurate constellation fidelity. However, STFT-GAN holds promise for waveform generation in experimental interference testing, *e.g.*, Ref. [6], where it is crucial to have good PSD fidelity to reflect in-band and out-of-band power characteristics and to capture time-frequency channel effects, but where an accurate signal constellation is not necessary. In future work, we plan to build on this investigation by developing generative models using real-world recordings of RF emissions for application to laboratory-based interference studies with real systems.

## 10 Appendix: WaveGAN

A state-of-the-art GAN that directly models time series with CNNs is WaveGAN [20]. In Sec. 8.1, we compared an implementation of WaveGAN, described below, against other GAN models for OFDM data. The original WaveGAN model produces single-channel audio waveforms of length 16384, using an architecture based on a 1-D flattened version of the popular DCGAN model for images [28]. WaveGAN attempts to widen its convolutional receptive field by modifying the DCGAN 5x5 kernels with 2x2 strides to 1-D kernels of length 25 with strides of 4 [20]. Also, in contrast to DCGAN, WaveGAN has no normalization layers, adds a fully connected layer to both models, and is trained with WGAN-GP loss.

In addition, WaveGAN adds a novel layer to the discriminator, called phase shuffle, which performs a random circular shift of a convolutional layer's output activation. Donahue *et al.* [20] found optimal results when the random circular shift was between -2 and 2 time steps. The aim of the phase shuffle operation is to make the model insensitive to the input waveform's phase, preventing so-called "checkerboard" artifacts, which manifest as spikes in the power spectrum [54].

Our slightly modified implementation of WaveGAN is shown in Tables 5 and 6, where *f* = 1, 2, or 4 for OFDM symbol lengths of 128, 256, and 512, respectively, and *n* is the batch size. Like the original WaveGAN model, our implementation used five convolutional layers for the generator and discriminator, but the dense layer was modified to support the lengths of our synthetic OFDM waveforms.

We attempted to implement phase shuffle for layers for which it was compatible, *i.e.*, layers with output dimension more than 4. However, we observed training instabilities with phase shuffle and therefore did not include it in our implementation. Note that the lack of phase shuffle may explain the spikes in the power spectra observed in Fig. 4.

Following the original WaveGAN training implementation [20], we trained the model with a 5:1 update ratio between the generator and discriminator and the Adam optimizer with *β*_1_ = 0.5 and *β*_2_ = 0.9. Otherwise, we followed the same training protocol described in Sec. 6. Namely, the learning rate was *α* = 10^-4^ for the generator and discriminator, the batch size was 128, and the model was trained for 500 epochs. Also, all target distributions were scaled between [-1, 1] using feature-based min-max scaling.

**Table 5:** WaveGAN generator architecture [ *f* = 1, 2, 4].

**Operation**	**Filter Shape**	**Output Shape**
*z* ∼ Uniform(-1, 1)		(*n*, 100)
Dense Reshape	(100, 1024 *f*)	(*n*, 1024 *f*) (*n*, 1024, *f*)
ReLU		(*n*, 1024, *f*)
Transpose Conv1-D (stride=4)	(25, 1024, 512)	(*n*, 512, 4 *f*)
ReLU		(*n*, 512, 4 *f*)
Transpose Conv1-D (stride=4)	(25, 512, 256)	(*n*, 256, 16 *f*)
ReLU		(*n*, 256, 16 *f*)
Transpose Conv1-D (stride=4) ReLU	(25, 256, 128)	(*n*, 128, 64 *f*) (*n*, 128, 64 *f*)
Transpose Conv1-D (stride=4)	(25, 128, 64)	(*n*, 64, 256 *f*)
ReLU		(*n*, 64, 256 *f*)
Transpose Conv1-D (stride=4)	(25, 64, 2)	(*n*, 2, 1024 *f*)
Tanh		(*n*, 2, 1024 *f*)

**Table 6:** WaveGAN discriminator architecture [ *f* = 1, 2, 4].

**Operation**	**Filter Shape**	**Output Shape**
*x* ∼ *G*(*z*)		(*n*, 2, 1024 *f*)
Conv1-D (stride=4) LReLU(*α* = 0.2)	(25, 2, 64)	(*n*, 64, 256 *f*) (*n*, 64, 256 *f*)
Conv1-D (stride=4) LReLU(*α* = 0.2)	(25, 64, 128)	(*n*, 128, 64 *f*) (*n*, 128, 64 *f*)
Conv1-D (stride=4) LReLU(*α* = 0.2)	(25, 128, 256)	(*n*, 256, 16 *f*) (*n*, 256, 16 *f*)
Conv1-D (stride=4) LReLU(*α* = 0.2)	(25, 256, 512)	(*n*, 512, 4 *f*) (*n*, 512, 4 *f*)
Conv1-D (stride=4) LReLU(*α* = 0.2)	(25, 512, 1024)	(*n*, 1024, *f*) (*n*, 1024, *f*)
Reshape		(*n*, 1024 *f*)
Dense	(1024 *f*, 1)	(*n*, 1)

## Supplemental Materials

⏺ Python code implementing our models and experiments is available at https://github.com/usnistgov/OFDM-GAN. Also, data files containing experimental results are available at https://doi.org/10.18434/mds2-2532.
